# Quality of care for older adults with chronic obstructive pulmonary disease and asthma based on comparisons to practice guidelines and smoking status

**DOI:** 10.1186/1472-6963-8-144

**Published:** 2008-07-08

**Authors:** Benjamin M Craig, Connie K Kraus, Betty A Chewning, James E Davis

**Affiliations:** 1 Health Outcomes & Behavior Program, Moffitt Cancer Center, Tampa, Florida, USA. Department of Economics, University of South Florida, Tampa, Florida, USA; 2 School of Pharmacy and Department of Family Medicine, University of Wisconsin, Madison, Wisconsin, USA; 3 School of Pharmacy, University of Wisconsin, Madison, Wisconsin, USA; 4 Department of Family Medicine, University of Wisconsin, Madison, Wisconsin, USA

## Abstract

**Background:**

The purpose of this study was to describe the prevalence of respiratory diseases in older adults and compare the demographic, health and smoking characteristics of those with and without these diseases. Furthermore, we evaluate the association between smoking status and patterns in health care and how concordant this care is with guidelines.

**Methods:**

Using a nationally representative sample of 29,902 older adults who participated in the Medicare Current Beneficiary Survey (1992–2002), we compared guideline recommendations on the treatment and prevention of chronic obstructive pulmonary disease and asthma with survey utilization data, including the use of bronchodilators, spirometry and influenza vaccine.

**Results:**

26% to 30% of older adults were diagnosed with or self-reported chronic respiratory diseases; however 69% received no pharmacological treatment and 30% of patients reporting use of pharmacological treatments did not receive short-acting bronchodilator inhalers. Current smokers appeared to receive significantly less care for respiratory diseases than non-smokers or former smokers.

**Conclusion:**

Disparities between recommended and actual care for older adults with chronic lung disease require further research. The needs of older adults with co-morbidities and nicotine addiction deserve special attention in care as well as guideline development and implementation.

## Background

Chronic obstructive pulmonary disease (COPD) is the fourth leading cause of death of adults in the world and a leading cause of death in people over the age of 45 in the United States [1]. The worldwide prevalence in 1990 was estimated to be 9.34/1,000 in men and 7.33/1,000 in women, but these estimates included all ages and may have underestimated the true prevalence in older adults. COPD causes a major financial burden to the healthcare system [2]. Under-diagnosis and under-treatment are associated with increased use of emergency department services due to exacerbations of the disease [3]. Implementation of practice guidelines, optimization of pharmacotherapy and reduction of risk factors, like smoking, have been shown to improve clinical and economic outcomes.

Likewise, asthma mortality continues to increase in the United States [4]. An analysis of asthma mortality between 1979 and 1996 showed that people over age 65 have the highest crude mortality rates from asthma. Similar to COPD, asthma is often under-diagnosed and under-treated in the elderly [5].

Practice guidelines for COPD and asthma have been available for a number of years [1,6-8]. Guidelines for the care of both COPD and asthma emphasize the importance of spirometry as an essential tool for the diagnosis and staging of severity [1,6,8]. Access to short-acting inhaled bronchodilators is recommended for persons with COPD and asthma across the spectrum of disease severity. Yet, evidence suggests that many patients do not receive recommended care [6,8]. Preventive interventions, such as immunizations and smoking cessation, are also promoted for this population. Tobacco use is recognized as a major risk factor for the development of COPD, and smoking cessation is acknowledged as a means to prevent disease progression [1,6]. Smoking also plays a role in exacerbating asthma [7,8]. Yet, smoking remains a common behavior among older adults with COPD and/or asthma [1,6-8].

The 1992–2002 Medicare Current Beneficiary Survey (MCBS) is the only nationally representative survey over this period that focused on the health and health care of older adults. Using this dataset, our aims are to determine the prevalence of COPD or asthma among older community-dwelling adults in the United States and their demographic, treatment and smoking characteristics. We will measure whether older adults with COPD or asthma receive any pharmacotherapy, and test the association between its delivery, medication type, and corresponding care. Furthermore, we will evaluate the quality of their care compared to guideline recommendations, with special attention to older adults who smoke.

## Methods

The Medicare Current Beneficiary Survey (MCBS) is unique in several respects. Most importantly, it is a continuous, panel survey of a nationally representative sample of the Medicare population. The survey applies overlapping cohort design and involves the over-sampling of those over age 85 [9]. In complement to the collection of survey data by face-to-face interviews over a four year period, Centers for Medicare & Medicaid Services (CMS) link the survey data to Medicare claims data over the survey years and all subsequent years. As of August 2005, CMS has released 1991 through 2003 survey data on 76,880 beneficiaries including 222,545 person-years of survey data and 254,554 person-years of claims data. Data from the *Access to Care *and *Cost & Use *components of the MCBS for the years 1991–2002 were combined and examined to improve understanding of access to and use of health care among older adults with COPD or asthma.

The CMS employed computer-assisted personal interviewing (CAPI) technology to collect survey data and referenced previous responses and claims to improve the accuracy of the survey data. Respondents were given containers to collect prescription receipts and medication bottles to assist respondent memory about prescribed medicine events. Interviews occurred in person three times per year, and respondents had to recall health care related events that occurred up to approximately four months before the interview. Response rates are 85% or more for initial community interviews; participation in subsequent rounds is 95% or more.

For this study, the sample selection criteria included that a respondent: (1) was surveyed between 1992–2002; (2) resided in the community setting in the continental U.S., Alaska or Hawaii; (3) survived the entire calendar year; (4) enrolled in Medicare Parts A and B for the entire year; (5) was age 67 years by December 31st of the survey year; and (6) had no participation in Medicare Plus Choice. CMS does not have claims data for the current or previous year for those beneficiaries who are younger than age 67, are not enrolled in Parts A and B, or participated in Medicare plus Choice; hence they were removed from the sample. The analytical sample for this analysis contains data on 29,202 respondents including 73,386 person-years of survey data since patients have multiple years in the sample.

Using the survey data, we analyzed patient demographic and socioeconomic data, as well as use of prescription medicines. Other than medication use, the survey variables represent fall survey responses. For example, smoking status represents point-in-time coverage during the fall interview of the survey year. Chi-squared and t-tests were applied to test the statistical significance of differences in respondent characteristics and delivery of health care by the presence of obstructive respiratory disease and pharmacotherapy (Tables 1 and 2, respectively).

**Table 1:** Characteristics of Older Adults With and Without Obstructive Respiratory Disease, 1992–2002

**Characteristics**	**No Obstructive ** **Respiratory Disease**	**Obstructive ** **Respiratory Disease**	**p-value**
Number of person-years	51861	21525	
Female, %	0.61	0.56	<0.01
Race, %			
White	0.88	0.89	0.73
Black	0.09	0.08	<0.01
Other	0.03	0.03	<0.01
Hispanic, %	0.04	0.04	<0.01
Income less than $25,000, %	0.66	0.72	<0.01
Education, %			
Less than High School	0.22	0.26	<0.01
High School	0.48	0.49	0.04
More than High School	0.29	0.24	<0.01
Drug Coverage, %			
None	0.47	0.42	<0.01
Medicaid	0.08	0.13	<0.01
Employer	0.32	0.31	0.28
Individual	0.09	0.09	0.99
Other Public	0.05	0.06	<0.01
Regional Characteristics, %			
Resides in MSA	0.60	0.60	0.6
Resides in Northeast	0.20	0.19	<0.01
Resides in Midwest	0.27	0.23	<0.01
Resides in South	0.39	0.42	<0.01
Resides in West	0.14	0.16	<0.01
Number of Chronic Conditions	1.14	1.36	<0.01
Chronic Conditions, %			
Hypertension	0.56	0.61	<0.01
Heart Condition	0.02	0.04	<0.01
Stroke	0.11	0.14	<0.01
Cancer	0.18	0.22	<0.01
Diabetes	0.16	0.18	<0.01
Arthritis	0.09	0.13	<0.01
Osteoporosis	0.01	0.02	<0.01
Fair or Poor Health Status, %	0.19	0.34	<0.01
Limitations in Activities of Daily Living, %			
No Limitations	0.73	0.61	<0.01
1 or 2 limitations	0.18	0.25	<0.01
3 or 4 limitations	0.06	0.09	<0.01
5 or 6 limitations	0.03	0.04	<0.01
Some or more difficulty walking 2 or 3 blocks or 1/4 mile	0.32	0.50	<0.01
Smoking History %			
None	0.47	0.31	<0.01
Former Smoker, %	0.45	0.53	<0.01
Current Smoker, %	0.08	0.16	<0.01

**Table 2:** Smoking History and Patterns in Health Care among Older Adults with Obstructive Respiratory Disease, 1992–2002

	**Older Adults with Obstructive ** **Respiratory Disease**			**Older Adults with Obstructive Respiratory ** **Disease & Treated with Pharmacotherapy**		
		
	**No ** **Pharmacotherapy**	Pharmacotherapy *	**p-value**	**No short-acting ** **inhaled** **bronchodilator**	**Short-acting ** **inhaled ** **bronchodilator †**	**p-value**
Number of person-years	14,894	6,631		1,995	4,636	
Annual use of health care, %						
Spirometry examinations	0.08	0.24	<0.01	0.21	0.26	<0.01
Pulmonologist visits	0.08	0.27	<0.01	0.25	0.29	<0.01
Influenza vaccination	0.67	0.77	<0.01	0.73	0.79	<0.01
Emergency room visits	0.20	0.26	<0.01	0.26	0.25	0.59
Number of physician visits	8.94	10.95	<0.01	11.30	11.03	0.99
Number of days in hospital	2.76	4.37	<0.01	5.48	3.86	<0.01
Respiratory medications, %						
Corticosteroid inhalers				0.23	0.35	<0.01
Xanthines				0.34	0.26	<0.01
Salmeterol				0.10	0.10	0.82
Leukotriene receptor antagonists				0.04	0.05	0.12
Oxygen				0.49	0.28	<0.01
Smoking History, %						
None	0.35	0.24	<0.01	0.28	0.21	<0.01
Former Smoker	0.49	0.61	<0.01	0.58	0.62	<0.01
Current Smoker	0.16	0.15	0.02	0.14	0.17	<0.01
Influenza vaccination	0.67	0.77	<0.01	0.73	0.79	<0.01

* Respiratory treatments include oxygen, ipratropium (Atrovent^®^) inhaler, ipratropium-albuterol (Combivent^®^) inhaler, xanthines, leukotriene receptor antagonists, short-acting beta antagonist inhalers, salmeterol (Serevent^®^), and corticosteroid inhalers.

† Short-acting inhaled bronchodilators (metered dose inhalers and nebulizer solutions) include: beta-agonists ipratropium bromide (Atrovent^®^), and the combination of ipratropium bromide and albuterol (Combivent^®^) inhalers.

& All health care measures represent utilization over a calendar year.

Diagnosis of COPD and asthma was measured using the presence of ICD-9 diagnostic codes on inpatient, outpatient, skilled nursing facility, hospice, home health agency, durable medical equipment, or physician claims, and through a survey question. For each survey year of data, the survey year's claims and previous year's claims were reviewed to identify respondents with a clinical diagnosis of COPD or asthma. These codes included COPD (chronic bronchitis–ICD-9 codes 490–491, emphysema–ICD-9 code 492, bronchiectasis–ICD-9 code 494, chronic airway obstruction–ICD-9 code 496) and asthma (ICD-9 code 493) [10]. In addition to clinical diagnoses through claims data, respondents were asked, "Has a doctor ever told you that you had emphysema, asthma, or COPD?" as a form of self-reported indication. Similar questions were asked about hypertension, heart conditions, stroke, cancer, diabetes, arthritis and osteoporosis, and these self-reported indications were aggregated to count an individual's chronic comorbidities. For example, the cancer indicator represents an affirmative response to the question, "Has a doctor ever told you that you had any kind of cancer, malignancy, or tumor other than skin cancer?"

The utilization of Medicare-covered health care services was measured using information taken from the claims data. Specifically, we identified (a) whether the respondent had any visits with a pulmonologist based on the presence of physician claims with specialty code 29; (b) any spirometry procedures based on the presence of physician or outpatient claims with Healthcare Common Procedure Code (HCPC) 94010, 94060, and 94375; and (c) any community-setting oxygen use based on durable medical equipment events during the survey year. Influenza vaccination for the previous winter was measured by self-report.

The utilization of outpatient prescription medications was measured based on survey information on prescribed medicine events. The prescribed medicine events were categorized using drug names and translated into the World Health Organization Anatomical Therapeutic Chemical (ATC) classification system [11]. We examined the prevalence of prescribed medicine events for all drugs for obstructive airway diseases (R03) including: corticosteroid inhalers (R03BA), xanthines (R03DA), selective beta-2 adrenoreceptor agonist inhalers (R03AC), leukotriene receptor antagonists (R03DC) and anticholinergic inhalers (R03BB). Over the period 1992–2002, anticholinergics agents included ipratropium and the combination of ipratropium with albuterol (Combivent^®^). Tiopropium (Spiriva^®^) was not available during this time.

Since guidelines for asthma and COPD recommended access to a short-acting inhaled bronchodilator for all patients with asthma and for symptomatic (mild-severe) patients with COPD, evidence of access to a short-acting inhaled bronchodilator was used as a surrogate for concordance with guideline recommendations for pharmacotherapeutic interventions [6,8]. Two other areas of overlap in guideline recommendations for asthma and COPD included use of spirometry and influenza vaccination. Spirometry was recommended by both guidelines for the diagnosis and staging of disease and as a means to monitor the disease progression and effect of treatment. Influenza vaccination was recommended by both guidelines in addition to the Center for Disease Control universal recommendation for influenza vaccinations for all patients over the age of 65 [6,8,12].

The study was approved by the University of Wisconsin institutional review board, which considered the study exempt due to its use of publicly available data sets (45 CFR 46.101(b)(4)).

## Results

### Health status of older adults with COPD or Asthma

**Figure 1:**
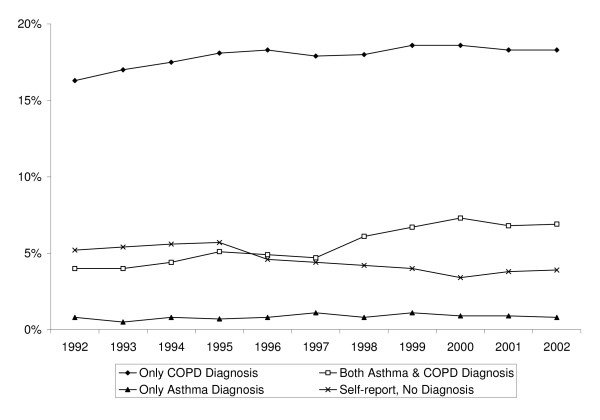
Prevalence of Obstructive Respiratory Diseases among Older Adults, 1992–2002.

The estimated proportion of older adults with COPD or asthma increased from 26% to 30% from 1992 to 2002 (Figure 1). The proportion of adults with only COPD (18%) claims or only asthma (<1%) claims has remained largely constant over the period 1992 through 2002. Over the late 1990's, the proportion of older adults with both asthma and COPD (5.6%) claims has increased, which may suggest that more adults with asthma are being treated as COPD patients. However, even with combining the asthma-only and asthma and COPD claims, the prevalence of asthma in this older population is still much lower than what would be expected in the general population.

The proportion of older adults with self-reported COPD or asthma and no claims has decreased. This last result, when combined with the evidence of little change in the relative prevalence of the respiratory diseases among older adults, may suggest that in the late 1990's diagnosed cases were more frequently treated, thereby rendering Medicare claims.

Compared to those without COPD or asthma, older adults with one of these conditions were more likely to have an annual income less than $25,000, to be less educated and to be enrolled in Medicaid (Table 1). In addition to their lower socioeconomic status, their health status was generally worse, including more chronic co-morbidities, lower self-reported health status, and greater limitations in activities of daily living. Fully 61% had hypertension, 22% had cancer, 18% had diabetes and 13% arthritis. They were also 56% more likely to report some difficulty walking two or three blocks compared to those without COPD or asthma.

### Patterns in health care

The following estimates in patterns of health care are reported in terms of annual prevalence rates. Disparities between ideal and real care exist across a range of indicators promulgated by practice guidelines for COPD and asthma. Each year about seventy percent of older adults diagnosed with COPD or asthma did not receive any respiratory medications (Table 2). Slightly less than 22% of older adults with COPD or asthma received short-acting bronchodilator inhalers. A smaller minority (13%) of older adults with either COPD or asthma received one or more spirometry examinations during the year. Lastly, 72% of older adults with COPD or asthma received influenza vaccinations recommended in guidelines.

Since there is no diagnostic severity index in the dataset to evaluate the intensity of treatment provided, it is useful to examine a subset of individuals whose care patterns suggest moderate to more severe disease. We examined the subset of older adults with COPD or asthma who had received pharmacotherapy (Table 2). Consistent with the possibility they had more disease severity, the data indicate this group experienced more emergency room visits, physician visits and days in the hospital than did their counterparts who did not receive pharmacotherapy for COPD or asthma.

Among this subset of older adults who received respiratory medications for COPD or asthma, we again see disparities between practice guidelines and care in practice, despite the fact they used significantly more preventive and specialty services. (Table 2) Annually, thirty percent did not receive any short-acting bronchodilator inhalers. Although spirometry is important for the diagnosis and grading of severity for both diseases, only about a quarter of this subgroup received spirometry examinations annually. Influenza vaccinations are recommended each year for all older adults, especially those with respiratory conditions, but about one fifth of this subset did not receive influenza vaccinations.

It is of interest to note, those who did receive short-acting bronchodilator inhalers, as recommended in the existing guidelines, had fewer days in the hospital. Adults treated with short-acting bronchodilator inhalers were also more likely to receive corticosteroid inhalers, and less likely to receive xanthines and oxygen.

### Smoking history and patterns in health care

A substantial portion (16%) of the older adults with asthma or COPD continued to smoke, and the majority (53%) were former smokers (Table 1). Compared to high school graduates with income over $25,000, current smokers were more likely to represent persons with lower incomes. Current and former smokers were more likely to receive respiratory medications, particularly short-acting bronchodilator inhalers, than adults who never smoked (Table 2). This association might be related to disease severity.

We next examined the association between smoking history and health care service use, as well as the relationship between pharmacological treatments and health care service use (Table 3). To do this, we estimated the odds and rate of service use controlling for demographic, socioeconomic, health, regional and time characteristics. Among those with asthma or COPD, the odds of receiving a spirometry examination were 24% less for a current smoker (OR = 0.76) compared to a non-smoker or former smoker. The rate of physician visits was also 22% less (RR = 0.78). For each health service, current smokers were less likely to receive care than adults who never smoked or quit smoking. Overall, smokers are more likely to receive pharmacotherapy, but if they continued smoking, they were less likely to receive respiratory services such as spirometry examinations and pulmonologist visits.

**Table 3:** Associations between Smoking Status and Patterns in Health Care among Older Adults with Obstructive Respiratory Disease, 1992–2002*

	**Any Spirometry ** **examination**^&^	**Any ** **Pulmonologist ** **visits**	**Number of ** **Physician Visits**	**Any Emergency ** **room visits**	**Any Hospital Days**	**Number of ** **Hospital Days**
	OR (95% CI) †	OR (95% CI)	RR (95% CI)	OR (95% CI)	OR (95% CI)	RR (95% CI)
Smoking history						
None	1.00	1.00	1.00	1.00	1.00	1.00
Former smoker	1.00 (0.88–1.13)	1.12 (0.98–1.28)	0.97 (0.94–1.01)	1.06 (0.97–1.17)	1.10 (1.00–1.20)	1.04 (0.99–1.09)
Current smoker	0.76 (0.64–0.91)	0.76 (0.64–0.91)	0.78 (0.75–0.82)	0.87 (0.76–0.99)	0.81 (0.72–0.92)	0.91 (0.85–0.97)

* The base case represents a relatively health 70 year old male with obstructive respiratory disease, but no smoking history. This person resides in a northeastern city and has no drug coverage, over $25,000 in income, and a high school diploma.

† The logistic and negative binomial regression estimates are linearly adjusted for demographic, socioeconomic, health, region and time characteristics (See table 1). The number of hospital days is examined using a two-part model (i.e., logit and negative binomial) because of excess zeros in the count of hospital days.

& All health care measures represent utilization over a calendar year.

## Discussion

Standards of care for patients with asthma and COPD emphasize the role that bronchodilators play for control of symptoms [6,8]. All persons with asthma should have access to an inhaled beta-agonist for rescue from acute symptoms [8]. Likewise, short-acting inhaled bronchodilators continue to be recommended for symptomatic treatment of mild, moderate and severe COPD [1,6]. Yet, 69% of this population with diagnosed obstructive respiratory diseases received no respiratory medications at all and 30% of those who did receive respiratory medications did not receive any short-acting bronchodilator inhalers.

Half of the adults without inhaled bronchodilators received oxygen therapy, perhaps representing persons with severe COPD who may have benefited from use of these agents [6]. Among those without inhaled bronchodilators, over one-third received xanthines. Long-acting xanthines have benefit in the treatment of COPD and may be added to other controller medications for moderate or severe persistent asthma; however inhaled bronchodilators are recommended as the preferred agents in both conditions due to reduced toxicity [6,8].

Both the asthma and COPD guidelines recommended use of spirometry for diagnosis, grading severity and monitoring progression of disease and response to treatment [6-8], but it was used infrequently (13.3%) in this population. Our low numbers of persons with asthma may be a result of misdiagnosis as COPD because spirometry was not often used.

Unlike the recommendations for bronchodilators and spirometry, which are targeted for patients with COPD and asthma, the recommendation for influenza vaccine was universal for all older adults [12]. Despite this recommendation, 28% of this high risk population did not receive annual vaccinations.

Clearly, smoking is a grave problem among older adults with COPD or asthma. Our finding that current smokers receive less care than non-smokers or former smokers is both troubling and intriguing. Further, that 16% who continue is of concern.

In summary, this research found substantial gaps between treatment guidelines and their implementation with regard to drug therapy, spirometry, vaccinations and smoking cessation in this population of vulnerable older adults. These findings pose as many questions as they answer. The overarching question is why clinical guidelines for patients with obstructive pulmonary diseases are not being followed for older patients with respect to medication treatment, spirometry, vaccinations, and smoking cessation. We believe that contributing factors could relate to physicians, patients, and perhaps even the guidelines themselves.

Studies demonstrate low physician adherence to clinical guidelines [13,14]. Although internists in one study recognized the benefit of adhering to practice guidelines, many were concerned about their effects on physician autonomy and decision making, health care costs, and satisfaction with clinical practice [15]. Others identified lack of physician familiarity and, at times, agreement with guidelines [16]. The 2001 guideline for COPD suggested that some clinicians had a nihilistic approach to COPD care because of the limited success of primary and secondary prevention, the notion that COPD was a self-inflicted disease, and the paucity of good treatments available [6].

This line of reasoning suggests the value of interventions which are both physician and health system focused. Reminder systems, information resources for both providers and patients, as well as systematic feedback on the quality of care provided through report cards and Healthcare Effectiveness Data and Information Set (HEDIS) measures might also improve the delivery of care [17,18]. Although guidelines are distributed widely to physicians in practice, they receive little education or practice implementing the guidelines within the constraints of a busy practice. Thus there is little opportunity to apply them within the realities of more complicated patients with other concerns.

Patient preference and choice may also account for deviation from adherence to guidelines. Do some patients prefer to focus more on treatment of other morbidities or on other therapeutic interventions for COPD? Substantial literature suggests that the majority of patients with a serious illness do want to play some shared role in therapeutic decisions [19]. Further, the concept of evidence-based medicine includes a role for patient preferences [20]. Let's take one example with smokers to explore this possibility. There may be a subset of smokers who experience such nicotine addiction that quitting would be very difficult. Recognizing this challenge, some physicians may have come to respect their patients' choices to continue to smoke. Another possibility is that a subset of physicians has dismissed these patients because they failed to motivate behavior change. A third possibility is that some of these patients withdrew from care to avoid uncomfortable encounters with physicians who urged smoking cessation. Without further research on the actual encounters between patients and providers, it is difficult to answer the question about how much patient preference contributes to the deviations documented in our analyses. There is a need for more work, specifically on patient contributions to this finding.

A caveat regarding the practice guidelines themselves is that most guidelines come from research that does not include older adults, calling into question the generalizability to this population [21]. Further, there are also questions as to whether the COPD guidelines in particular are evidence based in terms of using outcomes that matter to patients [22]. There is a need for more randomized studies using patient-oriented outcomes in older adults with COPD. Careful thought is needed to incorporate patient quality of life priorities and therapeutic preferences in regimen decisions. Both of these issues could lead to decisions to deviate from conformity to guidelines.

Adherence to multiple guidelines for patients with comorbidities can have negative tradeoffs [21]. For example, in our dataset, 61% of the patients have hypertension and 22% have cancer. Strict guideline adherence could potentially result in overly complicated regimens with increased risk of drug interactions and lower quality of life for people with more than one condition. It may well be that physicians are making tradeoffs with patients' comorbidities and competing priorities in mind. In addition to promoting more discussion of how to handle these situations, there is a glaring need to include more people with multiple chronic conditions in clinical trials that ultimately generate clinical practice guidelines.

Given that smoking cessation is the most important intervention in the management of COPD [23], it is disturbing that 16% in this data set continue to smoke. There is evidence that practitioner concern about harming the doctor-patient relationship may deter physicians from making smoking cessation interventions [24]. However, in at least one study when physicians are given written suggestions and tear off sheets for patients, physicians spoke with their patients about smoking cessation more often. As noted by other researchers in this area, we need more research on smoking cessation methods for older adults with COPD, specifically to be sure that smoking cessation guidelines are generalizable to this population who may have more difficulty quitting [25].

Ultimately, we need more primary research with older adults to complement our analysis of this unique, large dataset. This group of older patients has not received the intensity of research directed at younger populations with asthma and COPD. As a result, clinicians and patients alike are working with incomplete information needed to address complex decision-making. Especially given a movement to reward providers for adherence to practice guidelines, it is vital that we revisit the care of older adults. Basic science is needed to sort out optimal treatment strategies for patients who have comorbidities. More research is also needed to evaluate the complex decision making by patients and providers to understand the integration of population-based guidelines into daily practice.

## Limitations

The challenges involved in the study of older adults with obstructive lung diseases reflect the concerns relating to prevention, treatment and control of respiratory disease. To identify those with obstructive lung diseases, we rely on self-reported and claims-based indications of a clinical diagnosis, instead of medical record data. Self-reported measures may under-represent the prevalence of the disease due to recall bias, or may over-represent prevalence due to a misunderstanding of the survey question. We recognize that some affirmative responses to questions like "Did you have a flu shot for last winter?" may over-report use due to social desirability bias, and that claims-based measurement has also been shown to under-report the prevalence of asthma, and to over-report the prevalence of COPD [26]. Further measurement error may be related to shifts in coding practices and raised awareness over time. Although there are alternative measures for the identification of disease and stage, these measures are not feasible for a large epidemiologic study. We recognize that the self-reported and claims-based measures of clinical diagnosis have limitations, and have attempted to interpret the results accordingly.

The sample excludes those who were not enrolled in the traditional Medicare program for the full calendar year, and is not generalizable to the entire community-dwelling population of older adults. This exclusion was necessary to control for observation bias in the claims and prescribed medicine event data. For example, adults who recently enrolled in Medicare have missing claims data during the initial portion of the calendar year before their enrollment. Similarly, those who drop out of traditional Medicare, either through death, enrollment in Medicare Plus Choice, or disenrollment from Medicare, also have missing claims data.

## Conclusion

Despite the proliferation of numerous guidelines for the management of adults with obstructive respiratory diseases, we found major deviations in use of these guidelines. Substantial proportions of older adults with COPD or asthma do not receive a short-acting inhaled bronchodilators, infrequently used spirometry, fail to be vaccinated against influenza, and continue to smoke. Disparities between actual care and care recommended in guidelines suggest that more attention to older adults with lung disease is needed in both research and treatment. The special needs of older adults with comorbidities and long histories of nicotine addiction deserve special attention in guideline development and implementation. Reasons for less than optimal care need to be further explored.

## Competing interests

The authors declare that they have no competing interests.

## Authors' contributions

BMC–concept and design, acquisition of data, analysis and interpretation of data, drafting of manuscript, critical revision and statistical analysis. CKK–concept and design, analysis and interpretation of data, drafting manuscript, critical revision, and administrative/technical support. BAC–concept and design, analysis and interpretation of data, drafting of manuscript, critical revision, and administrative/technical support.

JED–analysis and interpretation of data, critical revision of the manuscript, and administrative/technical support. All authors have read and approved the final version of the manuscript.

## Pre-publication history

The pre-publication history for this paper can be accessed here:


